# Supplementary material for: Comparative genomics provides new insights into the diversity, physiology, and sexuality of the only industrially exploited tremellomycete: Phaffia rhodozyma
Source: BMC Genomics. 2016 Nov 9;17:901. doi: 10.1186/s12864-016-3244-7 (PMC5103461; doi:10.1186/s12864-016-3244-7)
Supplement: Additional file 6: — List of orphan genes with links to PFAM (related to Additional file 1: Table S1). (ZIP 1428 kb) [file 12864_2016_3244_MOESM6_ESM.zip › PFAM_UNIQUE/G02711_P.html]

#                                                                            --- full sequence --- -------------- this domain -------------   hmm coord   ali coord   env coord
  
# target name        accession   tlen query name           accession   qlen   E-value  score  bias   #  of  c-Evalue  i-Evalue  score  bias  from    to  from    to  from    to  acc description of target
  
#------------------- ---------- ----- -------------------- ---------- ----- --------- ------ ----- --- --- --------- --------- ------ ----- ----- ----- ----- ----- ----- ----- ---- ---------------------
  
G02711\_P             -            109 DUF4196              PF13846.1    112    0.0021   17.0   3.5   1   1   4.1e-07    0.0024   16.8   3.5    55   101    47    93    41   105 0.85 - Domain of unknown function (DUF4196)  
G02711\_P             -            109 DUF4482              PF14818.1    141     0.067   12.5   0.7   1   1   6.5e-05     0.077   12.3   0.7    65   114    37    86    27   106 0.83 - Domain of unknown function (DUF4482)  
G02711\_P             -            109 Period\_C             PF12114.3    195     0.073   11.3   0.8   1   1   0.00012     0.081   11.2   0.8    39    83    39    81    25   100 0.81 - Period protein 2/3C-terminal region  
G02711\_P             -            109 SR-25                PF10500.4    225       4.8    5.2   9.1   1   1     0.042       5.9    5.0   9.1    24    66    46    88    34    95 0.58 - Nuclear RNA-splicing-associated protein
